# Simultaneous bioconversion of lignocellulosic residues and oxodegradable polyethylene by *Pleurotus ostreatus* for biochar production, enriched with phosphate solubilizing bacteria for agricultural use

**DOI:** 10.1371/journal.pone.0217100

**Published:** 2019-05-16

**Authors:** Diana A. Moreno-Bayona, Luis D. Gómez-Méndez, Andrea Blanco-Vargas, Alejandra Castillo-Toro, Laura Herrera-Carlosama, Raúl A. Poutou-Piñales, Juan C. Salcedo-Reyes, Lucía A. Díaz-Ariza, Laura C. Castillo-Carvajal, Naydú S. Rojas-Higuera, Aura M. Pedroza-Rodríguez

**Affiliations:** 1 Laboratorio de Microbiología Ambiental y de Suelos, Unidad de Investigaciones Agropecuarias (UNIDIA), Departamento de Microbiología, Facultad de Ciencias, Pontificia Universidad Javeriana, Bogotá, D.C., Colombia; 2 Laboratorio de Biotecnología Molecular, Grupo de Biotecnología Ambiental e Industrial (GBAI), Departamento de Microbiología, Facultad de Ciencias, Pontificia Universidad Javeriana, Bogotá, D.C., Colombia; 3 Laboratorio de Películas Delgadas y Nanofotónica, Departamento de Física, Facultad de Ciencias, Pontificia Universidad Javeriana, Bogotá, D.C., Colombia; 4 Laboratorio Asociaciones Suelo, Planta Microorganismos (LAMIC), Grupo de Investigación en Agricultura Biológica, Departamento de Biología. Facultad de Ciencias, Pontificia Universidad Javeriana, Bogotá, D.C., Colombia; 5 Facultad de Ciencias, Universidad Anahuac de México, México, D.F., México; 6 Facultad de Ciencias, Fundación Universitaria Agraria de Colombia, Bogotá, D.C., Colombia; Gifu University, JAPAN

## Abstract

A simultaneous treatment of lignocellulosic biomass (LCB) and low density oxodegradable polyethylene (LDPE_oxo_) was carried-out using *Pleurotus ostreatus* at microcosm scale to obtain biotransformed plastic and oxidized lignocellulosic biomass. This product was used as raw matter (RM) to produce biochar enriched with phosphate solubilizing bacteria (PSB). Biochar potential as biofertilizer was evaluated in *Allium cepa* culture at greenhouse scale. Experiments including lignocellulosic mix and LDPE_oxo_ were performed for 75 days in microcosm. Biotransformation progress was performed by monitoring total organic carbon (TOC), CO_2_ production, laccase (Lac), manganese peroxidase (MnP), and lignin peroxidase (LiP) enzymatic activities. Physical LDPE_oxo_ changes were assessed by atomic force microscopy (AFM), scanning electron microscopy (SEM) and static contact angle (SCA) and chemical changes by Fourier transform infrared spectroscopy (FTIR). Results revealed *P*. *ostreatus* was capable of LCB and LDPE_oxo_ biotransformation, obtaining 41% total organic carbon (TOC) removal with CO_2_ production of 2,323 mg Kg^-1^ and enzyme activities of 169,438 UKg^-1^, 5,535 UKg^-1^ and 5,267 UKg^-1^ for LiP, MnP and Lac, respectively. Regarding LDPE_oxo_, SCA was decreased by 84%, with an increase in signals at 1,076 cm^-1^ and 3,271 cm^-1,^ corresponding to C-O and CO-H bonds. A decrease in signals was observed related to material degradation at 2,928 cm^-1^, 2,848 cm^-1^, agreeing with CH_2_ asymmetrical and symmetrical stretching, respectively. PSB enriched biochar favored *A*. *cepa* plant growth during the five-week evaluation period. To the best of our knowledge, this is the first report of an *in vitro* circular production model, where *P*. *ostreatus* was employed at a microcosmos level to bioconvert LCB and LDPE_oxo_ residues from the agroindustrial sector, followed by thermoconversion to produce an enriched biochar with PSB to be used as a biofertilizer to grow *A*. *cepa* at greenhouse scale.

## Introduction

Accelerated world’s population growth and industrialization generate considerable quantities of solid waste, where global composition of solid waste contains 46% organic residues, 17% paper, 10% plastic, 4% metal and 18% other non-specified waste [1]. Organic residues are composed by vegetable postcrop material, agroindustrial waste and organic and inorganic household waste. In many cities and companies solid organic waste and plastic are not properly utilized, are not sorted when disposed of, thus their final elimination takes place by incineration or ends up in landfills [1–3].

Plant and flower grower industries produce vast quantities of organic waste (bark, sawdust, leaves, stems, fragmented flowers, among others) containing high amounts of lignin, cellulose and hemicellulose [4–6]. In addition, these companies use low and high-density polyethylene (LDPE and HDPE) with different purposes: packing planting material, seedling rooting, mulching, greenhouse fabrication and wrapping of product [5,6].

Due to LDPE low biodegradability it becomes another environmental problem for these industries, so it is necessary to use [7,8] new forms of LDPE, such as oxodegradable (LDPE_oxo_) whose molecular structure ions or metal oxides have been incorporated that catalyze the polymer’s thermo/photooxidation once exposed to UV radiation or high temperatures [9,10].

Lignocellulosic residues and LDPE_oxo_ have different chemical structures, nevertheless both are transformed when chemical, physical and biological processes are utilized. Thus, sequential or simultaneous treatments can take place, when implementing biorefineries destined to sustain solid residue biodegradable conversion; and to certain extent non-biodegradable residues, as is the case for different types of plastics to obtain bioproducts [5,11]. These in turn can be used in other productive processes favoring sustainable natural resources use [12–14].

To carry out simultaneous bioconversion of LCB and LDPE_oxo_ different types of microorganisms can be used, by themselves, in co-cultures or in consortiums [15–17]. From an array of microorganisms studied, lignolytic white rot fungi are the most efficient to perform partial or total biotransformation of these types of solid residues [16–19] because of: (*i*) form networks of mycelia and produce exopolysaccharides that aid in colonization of both materials [6,20], (*ii*) produce an ample gamut of extracellular enzymes such as ligninases (E.C. 1.11.1.14), cellulases (E.C. 3.2.1.4) and hemicellulases (E.C. 3.2.1.), hydrolyzing hydrophilic and hydrophobic polymers generating smaller subunits [4,6], (*iii*) respond positively to addition of redox mediators to increase redox potential of enzymes, such as Lac (E.C. 1.10.3.2); allowing lignin non-aromatic fractions and LDPE_oxo_ aliphatic amorphous fractions to be oxidized [1] and (*iv*) oxidize aromatic and aliphatic subunits in lignocellulosic biomass and plastic in a non-selective manner, through non-enzymatic processes such as biological Fenton producing oxygen reactive species like hydroxyls [21,22].

Fungus use for simultaneous LCB and LDPE_oxo_ treatment can be effective, whenever physical and chemical factors are taken into account, such as temperature and moisture, pH, aeriation, particle size and C/N ratio [18,23,24].

On the other hand, to favor LDPE_oxo_ biotransformation physical or chemical pre-treatment must be carried-out [25,26]; particularly it has been observed O_2_ plasma discharge under low pressure conditions, facilitate LDPE biotransformation, as it increases its hydrophilicity, due to polar hydroxyl and carbonyl group formation. Plasma discharge generates an ablation on the material’s surface, helping with mycelium colonization exposing amorphous parts of the polymer [6,27].

Once simultaneous LCB and LDPE_oxo_ treatments are carried-out various bio-products are obtained, such as biotransformed plastic, CO_2_ and partially oxidized LCB. Low-density polyethylene can be reused in a new transformation process or recycled. Carbon dioxide is captured and redirected for bioconversion processes, such as microalgae production under autotrophic and mixotrophic conditions [28]. Last, oxidized LCB can be incorporated in another biorefinery stage requiring a thermochemical treatment for biochar production.

Biochar is a biocarbon variety that when applied by itself or enriched with beneficial microorganisms (PSB, nitrogen fixing bacteria, bacteria promoting plant growth, among others) is useful in microorganism support and improves the soil’s physical and chemical properties [29–31]. Hence, nutrient disposition is considerably favored, thus increasing greenhouse or field plant growth in comparison with soil or a mixture of soil with materials such as peat, coconut fiber, raw sawdust, rice husk or vermiculite, among others [29–31].

Development of environmental sustainable technologies is important to diminish agriculture industry negative impact. In countries such as Colombia, in the past 10 years plant and flower growing industries have worked in implementing good agricultural practices to decrease plastic use and control inadequate overexploitation and contamination, bringing about diminished edaphic biodiversity and decreased soil fertility resulting in loss of agricultural system productivity. In addition, generation of lignocellulosic residues or photobiomass is considered another critical issue for these industries, since residues are generated in enormous quantities. Their accumulation has a negative impact on the environment and their disposal is costly, since residues cannot be easily biotransformed, hence alternatives must be sought. Therefore, the present work proposed to demonstrate through a co-metabolism process the feasibility to perform simultaneous bioconversion of lignocellulosic biomass and low density polyethylene employing *P*. *ostreatus* as a low specificity enzyme complex producer. In addition, this work demonstrated the possibility to employ the bioconversion product for thermic biochar generation with physical and chemical properties that allow for phosphate solubilizing bacteria immobilization and its use to grow *A*. *cepa* at greehouse scale.

Therefore, the objective of this study was to simultaneously treat LCB containing a mix of pine bark (PB), chopped paper napkins (CPN) and brewing yeast hydrolysate (BYH) and LDPE_oxo_ employing *P*. *ostreatus* at microcosm scale to obtain oxidized LCB and biotransformed plastic as RM to produce biochar enriched with PSB. Its potential use was evaluated as a biofertilizer in *Allium cepa* at seedbed scale.

## Materials and methods

### Fungus reactivation and propagation in liquid media

*Pleurotus ostreatus* was obtained from the bank of strains at the Pontificia Universidad Javeriana Laboratory for Environmental microbiology and soil, Bogotá, D.C., Colombia. *P*. *ostreatus* was reactivated in wheat bran extract agar (175 gL^-1^ wheat bran, 10 gL^-1^ glucose, 2 gL^-1^ yeast extract, 5 gL^-1^ peptone, 0.05 gL^-1^ MgSO_4_·7H_2_O, 0.076 gL^-1^ MnSO_4_·H_2_O, 0.1 gL^-1^ KH_2_PO_4_, 0.1 gL^-1^ chloramphenicol, 20 gL^-1^ agar-agar) and incubated for eight days at 28°C [6,32]. For pelleted biomass production wheat bran extract broth was employed (the same wheat bran media without the agar) in 250 mL Erlenmeyer flask with 130 mL media, inoculated with 10 wheat agar discs containing the fungus’ mycelium. Erlenmeyer flasks were incubated for 10 days at 28°C under agitation at 120 rpm. After incubation, culture was centrifuged at 9,790 x g Sorvall RC 6 plus for 10 minutes at 4°C, biomass was washed five times) the first four with 0.85% (w/v) saline solution and the last with a 0.625 gL^-1^ glucose and 0.050 gL^-1^ ammonium chloride solution.

### Pristine LDPE_oxo_ sheet oxygen plasma pre-treatment

To obtain pristine LDPE_oxo_ sheets commercial “plastic bags” were cut into (3.0 ± 0.1) cm x (1.0 ± 0.1) cm sheets. Each sheet was washed for two minutes with 99.8% v/v methanol (Merck) and dried at 14°C for 15 minutes. Subsequently they were treated with glow discharge plasma (O_2_, 3.0 x 10^−2^ mbar, 600 V, 6 min), [6].

### Simultaneous lignocellulosic biomass (LCB) and plasma treated LDPE_oxo_ transformation curves under microcosm system

Previous to biotransformation curves, a 2^3^ factorial design with three central points was performed to select plant dry matter proportion as part of LCB’s rich filling mixture for microcosm evaluation (S1 Supplementary Material). Response variables were colonization percentage (%), total organic carbon (TOC), organic matter (OM) and Lac, MnP, and LiP enzyme activities. Selection of the best mix was determined by ANOVA analysis and mean comparison using Design-Expert (Stat-Ease Inc. 2017. version 11.0. Minneapolis, MN: Stat-Ease) and SAS (SAS Institute 2017. version STAT 14.3. Cary, NC: SAS Institute) softwares.

Once the best filling mixture was selected, microcosm system set-up was performed in 750 mL glass bottles, with hermetic seal rubber stopper adapted for a “J” aeriation system incorporating a 0.22 *μ*m gage Millex-GP SLGP033RB syringe, a port for nutrient input and an outlet for gas exit, connected to a 0.4 N NaOH trap for CO_2_ capture. Each microcosm was continuously aeriated at a 135 mL min^-1^ ratio, employing a Xilong AP-005 (110 V-60 Hz) engine, [4]. To each microcosm a mixture composed of 24 g PB, 27 g CPN and 9 g BYH in addition to 3% moist *P*. *ostreatus* biomass, 0.2 mL g^-1^ nutrient solution (MnSO_4_ gL^-1^ 0.5 gL^-1^, FeSO_4_∙7H_2_O 0.1gL^-1^, ZnSO_4_∙7 H_2_O 0.1gL^-1^, CuSO_4_ 1.5gL^-1^, ABTS 0.1mM), were supplemented every eight days and five plasma treated LDPE_oxo_ sheets were added. Mean temperature incubation was 22°C, for a 75-day evaluation period. Once the experiments were initiated, in addition to air injection each unit was handled under non-aseptic conditions. Bottles were manually turned upside down to guarantee biomass growth, mixture homogeneity, and increase *P*. *ostreatus* surface contact with plasma treated LDPE_oxo_ sheets.

Each treatment (TM1) and controls (CM1, CM2 and CM3) were evaluated by triplicates. In addition, every 15 days samples were collected to analyze response variables associated with LCB and plasma treated LDPE_oxo_ sheets and their association with Lac, MnP, and LiP activities. An aliquot of biotransformed LCB was used to quantity response variables, the remainder was used for biochar’s production, characterization, and employment for greenhouse *Allium cepa* growth experiments. LDPE_oxo_ was used for plastic characterization, and CO_2_ was captured with a NaOH trap to determine its concentration by respirometry assays.

### Microcosm biotransformed LCB thermal post-treatment

Microcosm biotransformed LCB in addition contained *P*. *ostreatus* mycelia, due to fungus growth during microcosm assay. This mixture rich in lignin, cellulose, hemicellulose, yeast hydrolysate and fungus mycelia were identified as RM and employed for biochar (BC) production. Initially, RM was dried at 90°C for 24 h, and sieved to obtain an approximately 5 mm particle size.

For BC production, 100 g RM were placed in an aluminum tray and introduced in a muffle furnace (Labtech), where a 10° C min^-1^ heat ramp was applied until reaching 300° C, followed by 150 mL min^-1^ N_2_ injection. N_2_ addition was repeated after 30 minutes to produce BC300 under O_2_ reduced conditions for 1 hour [33,34].

For RM, as well as BC moisture percentage, pH and topographic analysis were performed through SEM coupled to EDS, [4,35,36].

### Biochar phosphate solubilizing bacteria enrichment and *Allium cepa* growth evaluation under greenhouse conditions

To enrich BC with PSB, (BC/PSB) first a biofertilizer was produced with *Pseudomonas* sp., *Serratia* sp., and *Kosakonia* sp. in MT11B media (5.0 g L^-1^ phosphate rock (Calboy), 2.5 g L^-1^glucose, 0.5 g L^-1^ BYH, 0.5 g L^-1^ (NH_4_)_2_SO_4_, 0.2 g L^-1^ KCl, 0.3 g L^-1^ MgSO_4_, 0.004 g L^-1^MnSO_4_ *7H_2_O, 0.0004 FeSO_4_ g L^-1^ and 0.2 g L^-1^ NaCl) at pH 7.0 ± 0.2 for 6 h at 30° C and 200 rpm. Phosphate rock composition was: 25% total phosphate (P_2_O_5_), 32% calcium (CaO), 14% silica (SiO_2_) and 0.5% aluminum (Al_2_O_3_), (http://www.calboy.co). Final, biofertilizer’s concentration was 10 colony-forming unit (CFU) mL^-1^ logarithmic units. Subsequently, 100 mL of biofertilizer were mixed with 100 g BC and manually homogenized for 5 minutes until BC was completely moistened with PSB, thus here referred to as BC/PSB initial (I) (BC/PSB/I). Following, BC/PSB/I was incubated for 24 h at 30°C to generate a secondary culture (BC/PSB/SC) to replace bacteria that could have been injured during formulation or bioenrichment protocol following methods under Colombian Patent No. 13094384, 2015-09-17 [37].

BC/PSB/I concentration determination was performed through the decimal dilution technique and modified agar SMRS-1 surface count (0.5 gL^-1^ (NH_4_)_2_SO_4_, 0.2 gL^-1^ KCl, 0.3 gL^-1^ MgSO_4_·H_2_O, 0.004 gL^-1^ MnSO_4_·H_2_O, 0.0004 gL^-1^ FeSO_4_·7H_2_O, 0.2 gL^-1^ NaCl, 10 gL^-1^ glucose, 0.5 gL^-1^ yeast extract) enriched with 5 gL^-1^ phosphate rock, In addition, pH was determined following the protocol indicated by the Colombian technical Standard 5167 [35].

Five seeds of *A*. *cepa* were seeded buried at 3 cm depth and covered with the same material. Containers were maintained under greenhouse conditions (15°C with 12 h light/dark cycles, watering every 8 days) for five weeks. At the end of the growth (*n* = 5) plant response variables were quantified fresh weight (g) and height (cm). For solid samples PSB count and pH were determined [38].

### Biological, chemical and physic-chemical parameter quantification

To determine Lac, MnP, and LiP enzyme activity in microcosm, the sheets were removed, all filling material was mixed and 4 grams were extracted for solid-liquid extraction with addition of 50 mM sodium acetate buffer (pH 5.0 ± 0.2) and 0.01% Tween 80 (v/v) at a 1:5 ratio. Extraction was maintained under agitation at 200 rpm for 5 h. The mixture was filtered through Whatman No. 3 filter paper and centrifuged at 8,000 x *g* for 15 minutes to obtain and extract, where enzyme activities were evaluated [4].

Laccase activity (EC. 1.10.3.2) was quantified following a reported methodology [39] using 5 mM ABTS as substrate. One Lac unit is defined as the quantity of enzyme required to oxidize 1 μmoL ABTS in 1 minute. For MnP (EC. 1.11.1.13) activity 2,6 dimethoxyphenol in sodium acetate buffer (100mM) was used as substrate. MnP activity is defined as the quantity of enzyme required to oxidize 1 μmoL 2,6 dimethoxyphenol per minute [40]. LiP activity was determined by the oxidation of 2.7 mM veratryl alcohol. One unit of enzyme activity (U) is equivalent to the quantity of enzyme required to oxidize 1 μmol veratryl alcohol per minute [39,41].

pH and moisture percentage were determined following the methodology reported in Colombian Technical Standard 5167 of 2011 [42]. CO_2_ production was determined by the respirometry method. An acid-base titration was carried out, where produced CO_2_ reacted with 0.4 N NaOH [43].

Total organic carbon and OM content were assayed following the AASHTO T 267–86 protocols [44]. On the other hand, lignin was first extracted by performing a 0.5 M KOH lignin extraction, and then quantified employing the Tannin/Lignin test kit (HACH). Concentration was evaluated in HACH DR 2800 spectrophotometer at 700 nm [45]. To calculate E4/E6 ratio and determine polymerization level the same alkaline extracted sample was used, and absorbance were read at 465 nm and 665 nm, respectively. The E4/E6 ratio is calculated by dividing absorbance at 465 nm by absorbance at 665 nm [45,46]. To calculate C/N ratio samples were collected at day 0 and day 75 to determine nitrogen concentration and calculate C/N ratio by the Kjeldahl Bremmer method [47].

LDPE_oxo_ hydrophobicity change was determined in triplicate by the previously reported SCA method [48] and adjusted [6]. To define sheet surface roughness, sheets were observed through an atomic force microscope on contact mode (Nanosurf easyscan 2). Condition parameters for acquiring measurements were: size: 61.8 μm, set point: 50 nN; P-Gain: 1000; I-Gain: 100; D-Gain: 0 [49]. Each determination was performed in triplicate.

Pristine or plasma treated LDPE_oxo_ topography, as well as *P*. *ostreatus* material colonization was analyzed by SEM (Jeol JSM 6490LV). Specimens were first coated with a thin layer of gold under vacuum conditions with a Denton Vacuum sputter coater Desk IV. Specimens were examined using the following configurations: an accelerating voltage between 20 kV– 30 kV, SEI (Secondary Electron Image) signal and direct magnification at 500 and 6,500 X. To reveal the material’s chemical mapping, the same equipment was used to perform an EDS, [6]. This service was provided by Universidad de los Andes (Bogotá, D.C., Colombia).

To identify functional chemical groups FTIR analysis was performed using a Shimadzu IR Prestige-21 spectrophotometer. FTIR configurations were: Measurement Mode: % transmittance, Apodization: Happ-Genzel, No. of scans: 20, Resolution: 4.0, Range (cm^-1^): 400–4000 [6]. From spectrum collected data, carbonyl (*Ico*) [50] and vinyl (*Iv*), [51] indices were obtained.

### Statistical analysis

Microcosm experiment results, biochar production, and *Allium cepa* assays were first evaluated for parametric statistic compliance assumptions by performing normality tests (Shapiro-Wilks) and homogeneity of variance (Levenne). using SPSS software (IBM Corp. 2013. IBM SPSS for Macintosh version 22.0. Armonk, NY: IBM Corp). To determine statistical differences among treatments ANOVA was used with Tukey *post-hoc* test.

## Results

### LCB and previously plasma treated LDPE_oxo_ simultaneous transformation curves in microcosm system

*P*. *ostreatus* microcosm set-out for lignocellulosic material and LDPE_oxo_ sheet biotransformation (S2 and S3 Tables). From all response variables analyzed among treatments based on ANOVA and mean comparisons, colonization percentage and enzyme activities were prioritized, since colonization and high enzyme activities can favor LCB and LDPE_oxo_ biotransformation. For colonization percentage interaction among the three factors PB, CPN and BYH was significant (p = 0.0041) with a 28% contribution percentage. Regarding enzyme activities, different effects were observed depending on factors. Therefore, interaction among ABC factors was selected, with *p* values of 0.0041, 0.0489 and 0.0452 for colonization percentage, MnP and LiP activities, respectively. With reference to mean comparison among treatments, T8 was selected (24 g PB, 27 CPN and 9 g BYH) to carry on with microcosm, since at 10 days of evaluation it presented significant differences among treatments for: colonization percentage (99%), (*p* = 0.0048) and enzyme activities of 380, 167 and 14280 U Kg^-1^ (*p* = 0.0043, p = 0.0039 and *p* = 0.0026), (S4 Table), for Lac, MnP y LiP, respectively.

**Fig 1:**
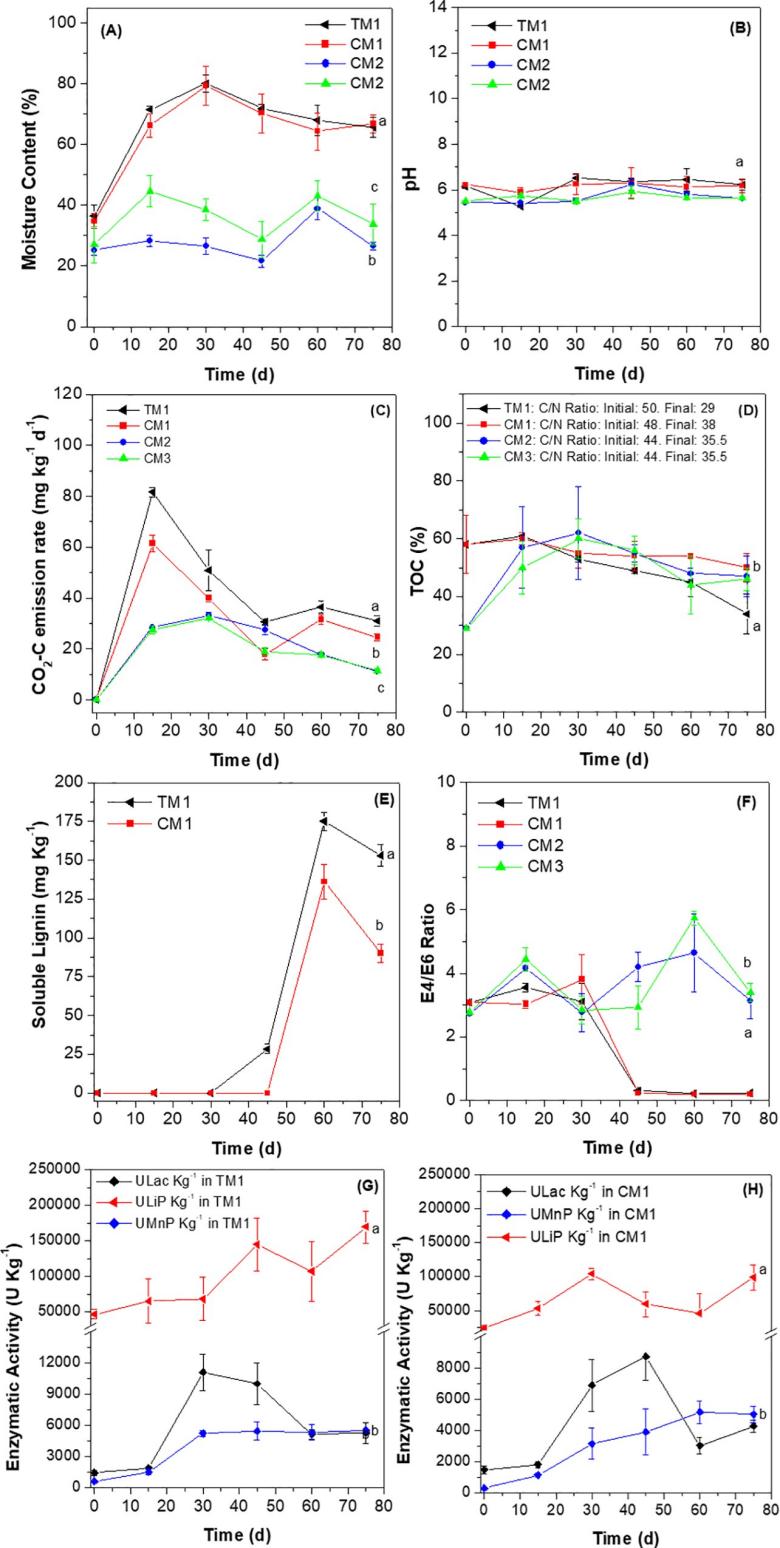
Microcosm analysis along 75 days. **(A)** Moisture percentage. (**B**) pH. (**C**) CO_2_-C emission rate (mg Kg^-1^ d^-1^). (**D**) Total organic carbon (TOC) percentage. (**E**) Lignin content (mg Kg^-1^). (**F**) E4/E6 ratio. (**G**) and (**H**) Lac (U Kg^-1^), LiP (U Kg^-1^) and MnP (U Kg^-1^) lignolytic activities. PB, CPN and BYH mixture. PO: *P*. *ostreatus*. TM1: Mix + PO + LDPE_oxo_. CM1: Mix + PO. CM2: Mix + LDPE_oxo_. CM3: Mix. Results presented correspond to mean of three replicas.

In microcosm experiments system initial moisture oscillated between 36 and 25%, where TM1 (PB + CPN + BYH + LDPE_oxo_ + *P*. *ostreatus*) and CM1 (PB + CPN + BYH + *P*. *ostreatus*) microcosm with *P*. *ostreatus* moist biomass had the highest moisture percentage. In these experimental units a gradual moisture increase was observed, until reaching at day 30 maximum values of 80 and 79%, followed by a decrease to end up at 65 and 66% moisture on day 75 of the process. These percentages were significantly higher (TM1: *p* = 0.002 and CM1: *p* = 0.023) when compared with CM2 (PB + CPN + BYH + LDPE_oxo_) and CM3 (PB + CPN + BYH) (26 and 33%), which could be attributed to supplementation with a nutrient solution every eight days. In addition, *P*. *ostreatus* biomass growth could have contributed to differences in moisture, since mycelium can retain up to 50–75% moisture when in solid culture (Fig 1A). For treatments and controls pH was below 6.5 ± 0.2 and for TM1 and CM1 a slight pH decrease as a function of time was observed, where 5.3 ± 0.2 was the lowest value at day 15 for TM1, subsequently increasing, but never above 6.5 ± 0.2. For controls pH ranged between 5.4 ± 0.2 and 5.6 ± 0.2 (0 and 75 days), (Fig 1B).

*P*. *ostreatus* inoculation and growth favored CO_2_-C emission production kinetics, with significantly higher values for TM1 and CM1 in comparison with CM2 and CM3, respectively (TM1: 31 vs. CM2 11.1 mg Kg^-1^ d^-1^ p = 0.014 and CM1: 24.2 mg Kg^-1^ d^-1^ vs. CM3: 11.5 mg Kg^-1^ d^-1^ *p* = 0.011). CM2 and CM3 lower CO_2_ emission production could be associated with abiotic factors or presence of accompanying microorganisms, with decreased capacity to mineralize LCB and plasma treated LDPE_oxo_ (Fig 1C).

On the other hand, CO_2_ emission production as a microcosm biotransformation indicator was inversely related to a decrease in TOC percentage for TM1 and CM1 (ρ = -0.85, *p* < 0.026 and ρ = -0.81, *p* < 0.015) respectively, which was more efficient for experimental units containing *P*. *ostreatus* and plasma treated LDPE_oxo_. Initial percentage for TM1 treatment was 58% and gradually decreased to 34%, representing a 41% TOC removal at 75 day of treatment. A lesser decrease was observed for CM1 treatment inoculated with *P*. *ostreatus*, not containing plasma treated LDPE_oxo_ (14%). Total organic carbon was similar for controls without *P*. *ostreatus* and did not changed more than 10%. Additionally, total nitrogen percentage was determined to calculate C/N ratio at the beginning and at the end of the process. At the beginning they were 50 and 48% for TM1 and CM1, respectively. After 75 days of treatment they decreased to 42% for TM1 and 21% for CM1. C/N ratio for CM2 and CM3 controls were 44% and after 75 days they did not exceed 20% (Fig 1D).

Soluble lignin and E4/E6 ratio results, extracted in 0.5 M KOH solution are presented in Fig 1E and 1F. Under these conditions only soluble polymeric or aliphatic fractions are presented, and not total, since part of them remained tightly bound to LCB’s cellulose and hemicellulose. For TM1 and CM1 during the first 30 and 45 days no detectable increase in lignin concentrations was observed, suggesting this polymer was not used as substrate during this initial time. In contrast, other simpler compounds, such as cellulose from paper napkins, BYH and added glucose to nutrient solution were employed to initiate biotransformation and fungal biomass growth within the microcosm. Subsequently (day 45) a gradual increase in soluble lignin concentration was observed with final concentrations at day 75 for TM1 and CM1 of 153 and 90 mg Kg^-1^, respectively. These treatments were significantly greater compared with plasma treated LDPE_oxo_ sheets and *P*. *ostreatus* biomass (*p* = 0.0231). This increase suggested initiation of total LCB lignin biotransformation, thus augmenting soluble lignin fraction.

Concerning polymerization ratio between aromatic and aliphatic fractions (E4/E6) initial treatment values for treatment T1 and controls (CM1, CM2, and CM3) were very similar and oscillated between 2.8 and 3.1. A stark decrease in E4/E6 ratio was detected at 45 days of treatment, obtaining values of 0.2 for TM1, as well as CM1. These semiquantitative results suggest a greater release of high molecular weight compounds with higher level of aromaticity was observed. TM1 E4/E6 results are inversely associated with soluble lignin concentration after 45 days of treatment, since for this later variable an increase was observed (ρ = -0.90, *p* < 0.0013). Even though for CM2 and CM3 controls, a variation as a function of time from day 0 to 75 was presented, E4/E6 ratio did not decrease below 1.0, demonstrating compound release with greater aromaticity level or condensation could not take place. On the contrary, the tendency was similar to absorption at 465 nm as well as 665 nm (Fig 1F).

Relating to lignolytic activities (Lac, MnP and LiP), TM1 and CM1 tendencies were similar. Never the less, TM1 activities were significantly higher than CM1 control for Lac, MnP and LiP (*p* = 0.0078, *p* = 0.095 and *p* = 0.0013). For TM1 LiP and MnP increased as a function of time, possibly they were responsible for lignin’s aromatic and non-aromatic fraction oxidation. Hence, LiP activity was the highest among all three enzymes with a value of 169,438 U Kg^-1^ at day 75. MnP enzyme activity oscillated between 624 and 5,535 U Kg, where this later value was observed at day 75 (Fig 1G).

For TM1 Lac activity increased from day 15 to day 30, in contrast with peroxidase activities, its highest attained value was at day 30 with 11,089 U Kg^-1^ activity, followed by a decrease to end with an activity of 5,267 U Kg^-1^ (Fig 1G). In contrast, CM1 activities were lower compared with TM1 at 75 days obtaining values of 98,655, 5,023 and 4,273 U Kg^-1^ for LiP, MnP and Lac respectively. This difference could be associated with absence of plasma treated LDPE_oxo_ sheets, and to 2,2′-Azino-bis (3-ethylbenzothiazoline-6-sulfonic acid) (ABTS) redox mediator supplementation. This redox mediator increases laccase oxidation potential favoring non-aromatic lignin fractions and aliphatic type compounds with C-H bonds, such as those present in plasma treated LDPE_oxo_ sheets.

As lignocellulosic biomass transformation associated variables were analyzed, plasma treated LDPE_oxo_ changes such as SCA, roughness, FTIR, AFM and SEM were studied to evaluate both byproducts simultaneous effects. Table 1 illustrates LDPE_oxo_ SCA and roughness at day 75.

**Table 1:** Plasma treated LDPE_oxo_ sheet physical changes SCA, roughness and *Ico/Iv* indices after 75 days in the microcosm.

Microcosm	TM1			CM2		
	Pristine	Day 75	Change at 75 d (%)	Pristine	Day 75	Change at 75 d (%)
**SCA (°)**	86 ± 3	14 ± 6	**84**^**a**^	86 ± 3	28 ± 7	67^b^
**Roughness (nm)**	10 ± 1	13 ± 3	**30**^**a**^	10 ± 1	11 ± 1	10^b^
***Ico***	1.77	1.64	**8**^**a**^	1.77	1.72	3^b^
***Iv***	1.06	1.04	**2**^**a**^	1.06	1.05	1^b^

Results in bold with letter **a,** were significantly different (p < 0.05) related to results with letter b

The most significant response variable was SCA (p = 0.001), (Table 1), demonstrating after 75 days LDPE_oxo_ sheets introduced into TM1 microcosm presented an 84% SCA decrease, while those introduced into CM2 microcosm percentage was 67%. LDPE_oxo_ roughness presented a 20% difference between TM1 and CM2 (*p* = 0.00433). Carbonyl index (*Ico*) and Vinyl index (*Iv*) variations were 8% and 2% for TM1 and for CM2 of 2% and 1%.

**Fig 2:**
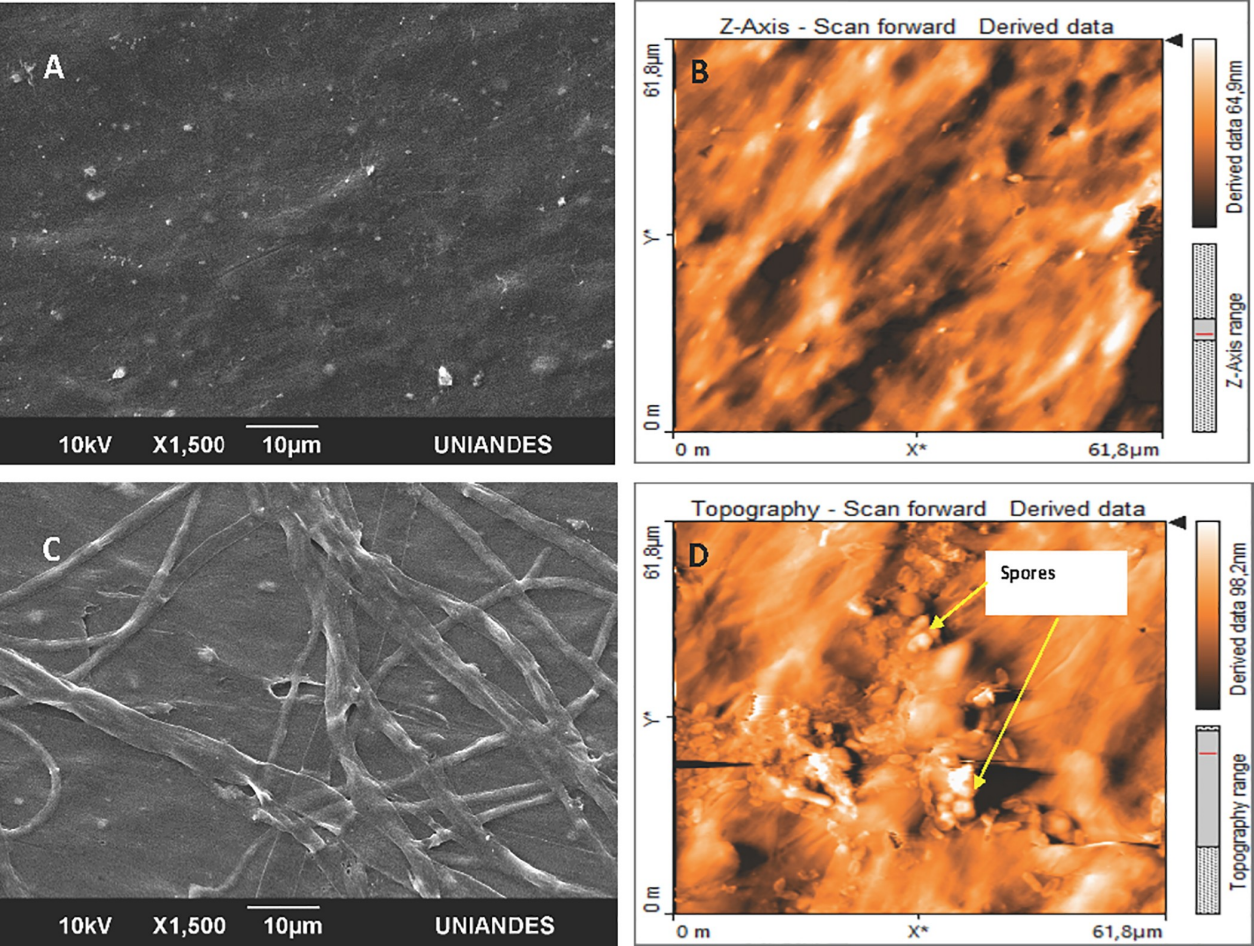
**SEM (A, C) and AFM (B, D) microscopic images. (A, B)** Pristine LDPE_oxo_. **(C)** LDPE_oxo_ SEM image after 75 days in microcosm. **(D)** LDPE_oxo_ AFM image after 75 days in microcosm.

To determine sheet roughness SEM and AFM microscopic images were used to determine sheet roughness. Pristine LDPE_oxo_ SEM and AFM images showed a homogenous and slightly rough surface (Fig 2A and 2B). Scanning electron microscopy image revealed *P*. *ostreatus* growth on the material’s surface (Fig 2C), where spores could be detected by AFM (Fig 2D).

**Fig 3:**
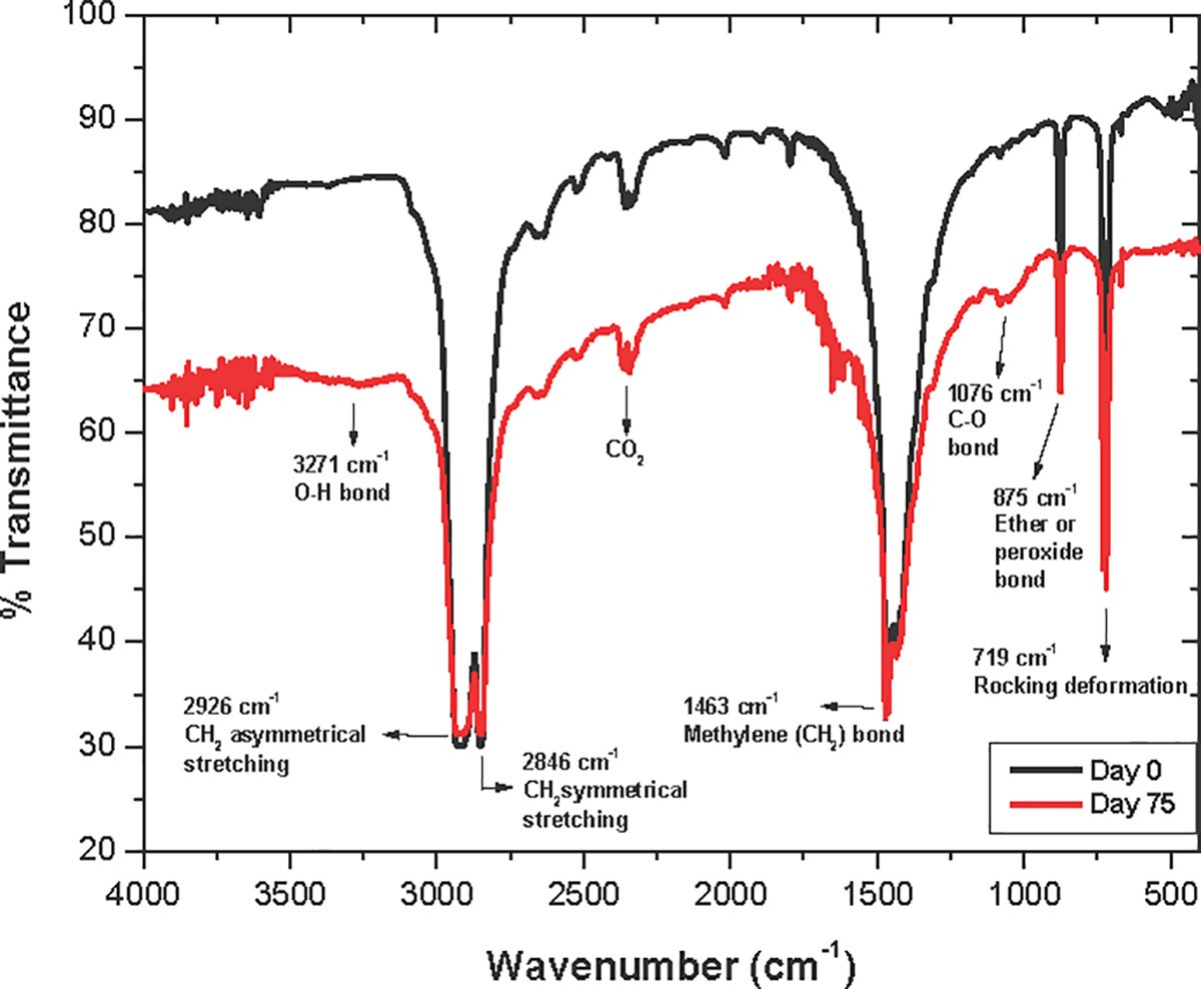
FTIR analysis for plasma treated LDPE_oxo_ sheets before incubation at day 0 (black line) and after 75 days of incubation in microcosm system (day 75: red line).

Fig 3 depicts FTIR obtained from LDPE_oxo_ after 75 days incubation in microcosm (red line). A slight increase in signals at 1,076 cm^-1^ and 3,271 cm^-1^, as well as 875 cm^-1^ were present indicating material oxidation process took place, corresponding to C-O and CO-H bonds, and ether bonds or peroxides respectively. In addition, a decrease in signals at 2,926 cm^-1^ and 2,846 cm^-1^ were observed, corresponding to CH_2_ asymmetrical and symmetrical stretching, respectively, associated with mass loss.

### Greenhouse biochar production: characterization and properties

The solid organic bioproduct obtained after 75 days of biotransformation in all microcosm systems (treatments and controls) was mixed to use as RM to carry out biochar (BC) production. Raw matter had a pH of 6.1 ± 0.2, consisted of 74% organic matter (OM), 56% TOC, 3.7% total nitrogen, a C/N ratio of 15, total bacteria count of 2.0 Log_10_ CFU g^-1^ and total fungi (*P*. *ostreatus* and yeasts) of 4.0 Log_10_ de CFU g^-1^ (Table 2).

**Table 2:** Raw matter proximate and element analysis for biochar produced and PSB formulation.

Sample	RM	BC	BC with PSB at initial formulation BC/PSB/I	BC with PSB at 30° C/24 h BC/PSB/SC
**Basic properties**				
pH	6.1 ± 1.1	7.3 ± 0.1	5.9 ± 0.2	7.0 ±1
OM (%)	74 ± 3	54 ± 3	82 ± 4	95 ± 6
TOC (%)	56 ± 4	31 ± 1	48 ± 3	53 ± 2
N (%)	3.7	ND	ND	ND
C/N ratio	15	ND	ND	ND
Total bacteria Count (Log_10_ CFU g^-1^)	2.0	< 2.0	9.5 ± 1.1	10.9 ± 1.7
Total fungi Count (Log_10_ CFU g^-1^)	4.0	< 2.0	< 2.0	< 2.0
Total PSB Count (Log_10_ CFU g^-1^)	< 2.0	< 2.0	9.5 ± 1.1	10.9 ± 2.3
*Pseudomonas* sp. Count (Log_10_ CFU g^-1^)	< 2.0	< 2.0	8.3 ± 1.4	8.7 ± 1.9
*Serratia* sp. (Log_10_ CFU g^-1^)	< 2.0	< 2.0	9.4 ± 1.8	9.3 ± 2.2
*Kosakonia* sp Log_10_ CFU g^-1^	< 2.0	< 2.0	8.6 ± 1.2	10.9 ± 1.4
**Proximate analysis**				
Yield (%)	-	53 ± 2	53 ± 2	53 ± 2
Moisture (%)	10 ± 2	0.1 0.02	63 ± 3	61 ± 2
Volatile Carbon (%)	85 ± 5	72 ± 4	81 ± 2	82 ± 5
Fixed Carbon (%)	12 ± 2	25 ± 3	17 ± 2	16 ± 4
Ash (%)	1.2 ± 0.6	2.9 ± 0.9	1.3 ± 0.3	1.9 ± 0.4
**Element analysis**				
C	44.5	87	54.7	53
O	50.8	12.5	32.8	33.5
H	4.7	0.5	12.5	13.5
Molar H/C ratio	0.105	0.0057	0.22	0.254
Molar O/C ratio	1.14	0.143	0.59	0.63

This mixed RM was subjecting to heating (300°C/1 hour) to obtain the BC, whose production showed changes for all parameters. pH increased to 7.3 ± 0.2, organic matter (OM) and TOC decreased to 54 and 31%, respectively. Additionally, all microorganism counts were below 2.0 Log_10_ CFU g^-1^. Regarding proximate analysis BC yield was 53% with a 0.1% moisture percentage. Carbon fractions were 72, 25, and 2.9% for the volatile and fixed fractions, and ash content, respectively. Therefore, produced BC was classified as Class II (organic carbon ≥ 30 and ≤ 60%), [52]. Carbon, hydrogen and oxygen element composition was evaluated in produced RM and BC. For carbon the thermochemical process generated an increase from 44.5 to 87%, and a decrease in oxygen from 12.5 to 0.5%. Moreover, degree of maturation and aromaticity was determined calculating H/C and O/C molar ratios, with values of 0.105 and 0.143, respectively (Table 2).

Once BC was produced and characterized it was enriched and formulated with PSB made up of *Pseudomonas* sp., *Serratia* sp., and *Kosakonia* sp. (unpublished results), and was named BC initial BC/PSB/I. The one obtained after 24 h of culture at 30° C was designated as secondary or (BC/PSB/SC). Both biochar (BC/PSB/I and BC/PSB/SC) were characterized to detect possible changes in basic properties, proximate analysis, element analysis and microorganism counts. For newly enriched BC (BC/PSB/I) a decrease in pH (5.9 ± 0.2), was observed, organic matter percentages and TOC (82 and 48%) increased, which could be associated with bacterial liquid culture supplementation. At 24 h of the BC/PSB/SC, pH increased to 7.0 ± 0.2, as well as OM and TOC (95 and 53%, respectively). These increases were associated with PSB within the BC and possible biofilm synthesis. Initial PSB counts at 24 h were high (9.5 and 10.9 Log_10_ CFU g^-1^, respectively). A 1.5 log unit increase suggests SC allowed for injured bacterial cell replication. Morphological counts were high and similar, oscillating between 8.3 and 10.9 Log_10_ CFU g^-1^, demonstrating formulation did not affect any bacteria conforming the phosphate solubilizing bacteria consortium (Table 2).

Proximate analysis results are presented in Table 2. BC yield did not change during formulation process, since it wasn’t submitted to any additional treatment, thus it remained at 53%. Moisture and volatile fractions increased with respect to BC/PSB/I, with percentages of 63 and 61, respectively and 81 and 82% for BC/PSB/SC. Increase was associated with moist PSB during the formulation process. In contrast, BC/PSB/I in comparison to BC without PSB, fixed fraction and ash content slightly decreased. For BC/PSB/I and BC/PSB/SC results were for fixed fraction 17% and 16%. For ash were 1.3% and 1.9%, respectively. Carbon element for BC300/PSB/I and BC300/PSB/SC decreased (54.7 and 53%, respectively) with respect to BC300 without PSB. Biochar acquired water under the aqueous base formulation process, resulting in greater proportion of H and O, as supported by H/O and C/O molar ratios, which were increased above 0.2, demonstrating a greater presence of polar groups and greater water content (Table 2).

**Fig 4:**
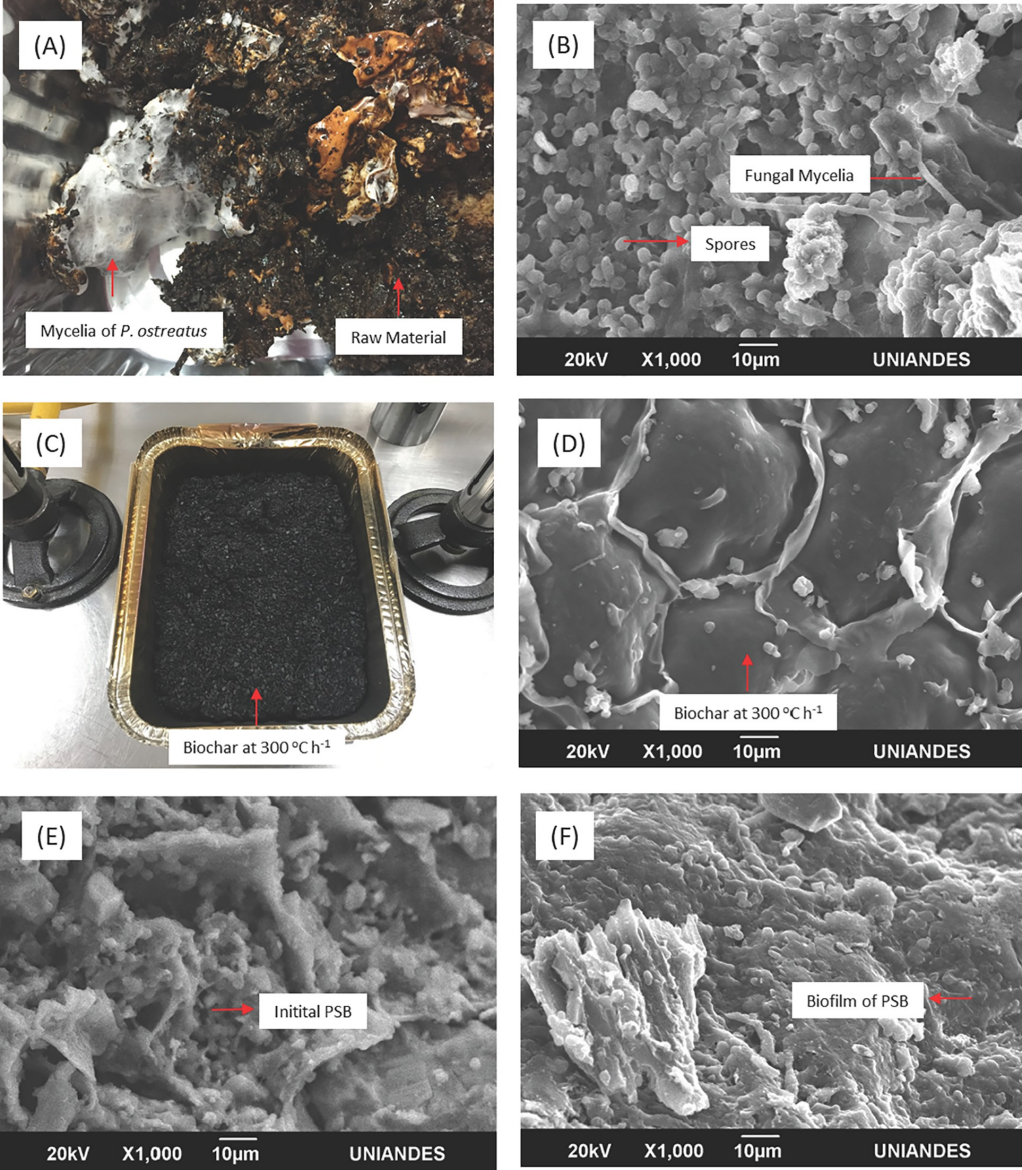
LCB and pretreated LDPE_oxo_ in microcosm. (A) RM. (B) RM SEM at 1000 x. (C) BC (D) SEM of BC. (E). BC/PSB/I. (F) BC/PSB/SC.

Raw matter was an LCB heterogeneous moist mix with different degrees of transformation and *P*. *ostreatus* mycelia (areas of white color of cotton-like texture Fig 4A). Raw matter SEM image analysis revealed *P*. *ostreatus*’ thin hyphae and sphere-shaped spores (Fig 4B). Upon BC production a more homogenous product was obtained, of black color and thick texture (Fig 4C). Smooth surface, panel-like structures with mycelia fragments or cell debris were observed on BC/PSB/I SEM images following 300°C treatment (Fig 4D). For BC300/PSB/SC formation of a uniform biofilm on BC’s surface was observed, which could be associated with bacteria growth after 24 h incubation at 30°C (Fig 4E and 4D).

### *Allium cepa* greenhouse growth assay

*A*. *cepa* growth (cm) and weight (mg) was favored by adding BC enriched with PSB, where the best treatment was T3 (5% BC enriched with PSB). After five weeks of evaluation height was 12 cm and weight 58 mg (*p* = 0.0010 and *p* = 0.0023). In contrast, T2 results were 7.8 cm and 38 mg and T1 6.1 cm and 35 mg. Observed results for controls (C1: only soil, C2: BC with PSB without soil, C3: only PSB, and C4: PSB without soil) were all lower compared with treatments (Table 3), thus demonstrating BC supplementation with PSB to soil could be a strategy enhancing *A*. *cepa* plant growth.

**Table 3:** Biochar and soil effect on *Allium cepa* growth. Greenhouse results of *Allium cepa* seed growth for five weeks under greenhouse conditions with different biochar concentrations and soil.

Code	Treatments	pH	Log_10_ CFU g^-1^	Fresh weight (mg)	Height (cm)
1	BC 1% (w/v) + PSB+ Soil	6.4 ± 0.1^c^	8.3 ± 0.2 ^**a**^	35 ± 3.5^b^	6.1 ± 0.3 ^b^
2	BC 2% (w/v) + PSB+ Soil	6.3 ± 0.2 ^c^	8.1 ± 0.2 ^**a**^	38 ± 1^b^	7.8 ± 0.3^b^
3	BC 5% (w/v) + PSB+ Soil	6.7 ± 0.6 ^c^	8.1 ± 0.8 ^a^	58 ± 1^a^	12 ± 0.4 ^a^
C1	100% soil	5.2 ± 0.2 ^d^	5.4 ± 1.2^c^	18.4 ± 4.9 ^d^	3.9 ± 0.2 ^d^
C2	BC at 100% (w/v) + PSB	8.0 ± 0.1 ^a^	6.8 ± 0.2 ^b^	25.7 ± 1.5 ^c^	5.7 ± 0.6 ^c^
C3	PSB only	7.0 ± 0.1 ^b^	5.1 ± 0.1 ^c^	21.3 ± 6 ^c^	4.2 ± 0.3 ^d^
C4	BC at 100% (w/v) w/out PSB	6.6 ± 0.1^c^	2.0 ± 0.1^e^	9.3 ± 2.9 ^e^	3.1 ± 0.5 ^e^

Means in a column followed by the same letter are not significantly different at p ≤ 0.05 by LSD, different letters mean statistical differences.

## Discussion

### Simultaneous LCB and plasma treated LDPE_oxo_ biotransformation in microcosm systems

Incinaration on landfill disposal has been for a long time the route for plastic residue disposal, such as LPDE. Incineration emits into the atmosphere toxic gases (Co_x_, No_x_; So_x_), increasing the greenhouse effect. In landfills, in layers below the cover vegetation and top soil, polymers biodegrade very slowly, due to oxygen and moisture restrictions. Biodegradation under anaerobic conditions produces CH_4_ (methane), a more potent gas compared with CO_2_, further increasing the greenhouse effect [53].

Another alternative for LDPE_oxo_ disposal and use of waste is to integrate it with other biotransformation technologies, such as industrial composting conditions (high temperatures, high moisture and oxygen levels), soil remediation and bioreactor (microcosm) use for solid LCB fermentation [5,53]; substantially differing from conventional practices including incineration and landfill disposal [17,52]. Because it is an aerobic process, degradation is faster, complete, and generates CO_2_ and biomass (living cells), [53]. Simultaneous treatment of LCB and plasma treated LDPE_oxo_ is a complex process, given chemical nature of both types of residues. However, if physical and chemical conditions are adjusted assisted by *P*. *ostreatus* biotransformation, it is possible to attain simultaneous transformation, without implying modifications at the same rate, and efficiencies, unless complete mineralization to CO_2_ is achieved [52,54].

Based on microcosm level obtained results *P*. *ostreatus* gradually biotransformed LCB under mesophilic aerobic conditions for 75 days. Among the intrinsic conditions evaluated, moisture percentage was not a biotransformation parameter. However, it was a critical variable to be controlled, since it determined substrate hydration, which helps to regulate oxygen content within the solid matrix, supporting mycelia growth and material’s surface colonization during the first days [55].

In the present study initial moisture for treatment and controls was low, never the less by progressive nutrient supplementation it was possible to adjust moisture percentage improving TM1 up to 80% and CM1 to 79% at day 30. Later they diminished to end with values reported for *P*. *ostreatus’* solid culture (50–75%), and different filling mixtures in composting processes degradation (60%), [24,55]. Additionally, *P*. *ostreatus* growth within TM1 and CM1 microcosms, also contributed to an increase in moisture percentage, since water can represent up to 50% of the fungus weight [55]. In contrast, no increase in moisture percentage was observed for controls without *P*. *ostreatus*, since they were only hydrated with nutrient solution and the quantity added was too low to generate an equal or higher percent in comparison with TM1 and CM1 (Fig 1A).

To initiate microcosms utilized material (PB+CPN+BYH) had different pH values: PB (5.5 ± 0.2), CPN (7.0 ± 0.2) and BYH (5.9 ± 0.2). Mix preparation gave an initial value below 6.5 ± 0.2, because PB and BYH had a greater initial influence on pH regulation, benefiting growth and *P*. *ostreatus* enzyme activity [18,55,56]. In regard to microcosm’s pH containing *P*. *ostreatus* biomass, pH reduction was associated with organic acid production given simple carbon sources, such as glucose, as well as cellulose’s and hemicellulose’s homo- and hetero-glycans [18,56]. During the last days of sampling pH increased to a value closer to the initial days, which could be accounted by ammonium production and volatilization because of BYH organic nitrogen source metabolism. It is known *P*. *ostreatus* can employ different sources of organic and inorganic nitrogen, where ammonium results as a product from the process of biotransformation in compost and edible mushrooms (Fig 1B), [24,55].

Progressive CO_2_ emission rate increase in TM1 and CM1, indicated biotransformation initiated at the expense of more easily biodegraded materials, *i*.*e*. paper napkin cellulose, glucose in nutrient solution, and carbon as part of the yeast hydrolysate. Because it is chemically less complex than PB, these compounds are the first to be hydrolyzed and used for mycelial growth, as was evidenced by an extensive hyphae network at the surface of the microcosm during the first days of the process. Toilet paper has been used as the carbon source to induce extracellular enzyme production and as initial fungal growth [27]. Our results are similar, since adding LDPE_oxo_ to LCB resulted in increased CO_2_ emission rate production. This was demonstrated with CM1 control composed of LCB and *P*. *ostreatus* biomass without any LDPE_oxo_ sheets, with significantly less production (*p* = 0.015), (Fig 1C). Quantified CO_2_ emission rate in CM3 and CM4 controls could be attributed to abiotic hydrolytic processes or by its accompanying microbiota. Other authors have also reported CO_2_ abiotic production. Some authors inoculated *Geobacillus* spp., in compost with plastic for 90 days, and observed absolute control had lower CO_2_ production in comparison with treatments [52].

Statistical analysis demonstrated CO_2_ emission rate production was negatively correlated with TOC percentage, as CO_2_ emission rate concentration increased and TOC percentage decreased (ρ = -0.85, *p* < 0.026 and ρ = -0.81, *p* < 0.015). However, for this variable two phases were detected. During the first 15 days a slow decrease was observed, suggesting LCB’s more stable fraction was unhydrolyzed (PB: lignin, cellulose and hemicellulose). Up to that time point certain fractions of total lignin had initiated depolymerization, in addition to bond hydrolysis among PB’s structural polymers. TOC percentage decrease was more evident for TM1after 30 days, likely due to alternations between labile (CPN, BYH and glucose) and stable carbon fraction biotransformation processes, once the delignification had initiated (Fig 1D), [18]. An appropriate C/N ratio is fundamental for this to take place, considering *P*. *ostreatus* requires available nitrogen to oxidize carbon fractions. For TM1, addition of CPN and BYH adjusted initial C/N ratio to 50/1. After 75 days of the process this ratio was reduced to 42% (TM1) and 21% (CM1), (Fig 1D). Even though, percentage reduction in C/N ratio were not high, results revealed biotransformation was carried out from C/N ratio, and solid fermentation could be prolonged in the microcosm.

Author’s suggest that using highly lignified materials in combination with other more biodegradable agroindustrial residues requires longer processing times for the C/N ratio to decrease below 15. Moreover, they can be considered as soil conditioners with a high degree of maturity [24,55,57]. Several authors also evaluated the effect of different waste materials to balance C/N ratio and increase biodegradability of forest residues in combination with plastic. In their “*in vitro*” study RM (36% (w/v) vegetable residues, 20% (w/v) rice, 29% (w/v) mature compost, 13% (w/v) sawdust as a bulking agent, and 2% (w/v) paper) were fermented with LDPE sheets and inoculated with *A*. *fumigatus*, *A*. *terreus* and *F*. *solani*. Under evaluated experimental conditions after 100 days of fermentation, these fungi decreased TOC by 42.31%, 21.2% and 8.13% respectively. Additionally, changes in LDPE sheets were evidenced suggesting biodegradation took place under solid fermentation conditions [19,57].

Other results that aid supporting different LCB carbon fractions (labile and stable) biotransformation were soluble lignin concentration and E4/E6 polymerization ratio. *P*. *ostreatus* lignin concentration was detected in TM1 at day 45 (28 mg Kg^-1^). This result supports the hypothesis that *P*. *ostreatus* first used more simple carbon sources, and on lignin it probably only carried-out modifications mediated by lignolytic enzymes that could be associated with partial oxidations of phenyl-propane, demethylations and hydrolysis of aliphatic chains [18,58–60]. Once depolymerization took place, PB delignification was evident, since soluble lignin concentration increased after 45 days and ended at day 75 with 153 and 90 mg Kg^-1^ for TM1 and CM1 respectively (Fig 1E).

For TM1 and CM1 E4/E6 polymerization level determination for the first 30 days, values were between 2.8 and 3.1, suggesting compounds with different degrees of aromaticity and aliphatic compounds were present (Fig 1F). Additionally, values below 1.0 could be associated with fractions with greater level of aromaticity and more condensed, such as humic and fulvic acids, indicators of a humus production process [61–63]. These results suggest that for TM1 and CM1 microcosms humus production process took place. High E4/E6 ratio during the early stages of organic matter degradation in soils of different uses, such as forests and coffee plantations [64], as that observed in this work at day 0 were observed. Moreover, in a caprine manure and shea-nut cake composting assay an E4/E6 ratio decrease from 4 to 2.68 in moist organic matter was observed [65]. The same behavior in pine sawdust compost has been observed, attributed to an increase in the number of condensed unsaturated aromatic rings, indicators of OM stabilization [66].

Lignolytic enzymes actively participated in LCB biotransformation process, yet their activity was distinct for each enzyme. During the entire process the enzyme with highest activity was LiP, since it is the first enzyme to initiate lignin modification given its superior redox potential compared with Lac (E0 = 1.2 V vs., 0.8 V, respectively). LiP can randomly oxidize the polymer’s phenolic and non-phenolic fractions (Fig 1G and 1H). Additionally, this enzyme can open aromatic rings to generate intermediate aliphatic structures (tricarboxylic acids) that are rapidly used for energy production [67].

Compared to Lac, MnP had a similar redox potential (0.8 V). However, in contrast to Lac as long as there is Mn^2+^ in the media, supplemented in nutrient solution, it can oxidize lignin aromatic and non-aromatic fractions (Fig 1G and 1H). According to several authors, under these conditions MnP can produce peroxyl and acyl type free radicals (low specificity intermediaries) that play a complementary role in lignin’s non-aromatic fraction oxidation [67,68].

Last, Lac activity was detected in TM1 treatment and its CM1 control. Nevertheless, TM1 activity was higher, demonstrating joint addition of LCB, ABTS redox mediator and plasma treated LDPE_oxo_ sheets could potentiate Lac activity (Fig 1G and 1H), [6,18,22,68,69].

LCB biotransformation and plasma treated LDPE_oxo_ could have taken place by combined action or synergy of a group of oxidative enzymes (ligninases) and hydrolytic enzymes (cellulases and hemicellulases), acting through a co-metabolic process, not only degrading their target substrates (lignin, cellulose and hemicellulose), but LDPE_oxo_ anthropomorphic generated contaminating compound, profiting on lignolytic enzyme low specificity [4,6,27,70]. In this work, addition of CPN as a carbon source favored synthesis of lignocellulosic enzymes of laccase type. Additionally, its action could have favored LDPE_oxo_ biotransformation through co-metabolism. Lac activity on LDPE_oxo_ has been reported when toilet paper was added as a co-substrate [27]. In addition, air was frequently administered to the microcosm system, promoting aerobic co-metabolism, which has as the following advantages: high degradation degree of an ample group of contaminants and low accumulation of toxic compounds [71].

Concerning plasma treated LDPE_oxo_ sheets, the most important change observed was TM1’s SCA decrease. In comparison with pristine LDPE a 72° decrease was observed, i.e. an 84% gain in hydrophilicity. This result reveals the synergistic effect plasma discharge and fungus enzyme activity have on the material’s oxidation. Additionally, it highlights microbial enzyme capacity to oxidize high molecular weight polymers, generating functional groups that increase hydrophilicity [72]. Moreover, if LDPE_oxo_ is surrounded by a moist environment (close to 70%, as was observed in the microcosm), it maintains its hydrophilicity, since an aqueous environment has affinity for polar groups and forces them to remain on the surface [73].

For CM2 with a 28 ± 7° SCA, a moderate recovery of post plasma hydrophobicity could have presented, allowing for non-oxidized low molecular weight chemical species to migrate from the material’s bulk to the surface, making it slightly hydrophobic [26]. In the present work a 75% hydrophobicity recovery for non-oxobiodegradable LDPE sheets under plasma discharge was observed after seven days [6]. In this study, CM2 LDPE_oxo_ sheet recovery was of 67% at day 75 (Table 1), suggesting employed microcosm maintained LDPE_oxo_ post-plasma hydrophilicity, enhancing microorganism growth and colonization on the material’s surface, thus its biotransformation.

*P*. *ostreatus* growth and colonization on plasma treated LDPE_oxo_ sheets was evidenced by an increase in the material’s roughness [(13 ± 3) nm] compared with control, as was observed in AFM images and confirmed by SEM images. Fungi secrete chitin and glycan facilitating adhesion to polyethylene surface, as well as hydrophobines that form a hydrophobic-hydrophobic interphase, aiding in hyphae penetration and mycelial network growth [6], thus increasing roughness. *P*. *ostreatus* capacity to grow on LDPE_oxo_ generating grooves, cracks and holes on polyethylene’s surface, due to mechanical action of hyphae apices has been described [6,27,74]. In the present work no cracks or grooves were observed on SEM images that would allow to evidence fungus mechanical action.

FTIR results revealed plasma treated LDPE_oxo_ biotransformation (Fig 3), where signals corresponding to C-O (1,076 cm^-1^) and C-OH (3,271 cm^-1^) bonds suggested material’s oxidation. Furthermore, variations in the signal at 2,928 cm^-1^, 2,848 cm^-1^ are associated with loss of mass [75]. During the 90-day process of LDPE_oxo_ biotransformation with TiO_2_ as a peroxidation promoting agent and *P*. *ostreatus*, signals at 3,500–3,000 cm^-1^ corresponding to hydroxyl (C-OH) group were described before [27], as observed in the present work. Presence of polar groups favored LDPE_oxo_ hydrophilicity (Table 1), [42,74], which allowed *P*. *ostreatus* to use the polymer as a substrate, and colonize it (Fig 2C and 2D).

In one study authors identified in LDPE_oxo_ treated with TiO_2_, UV radiation and heat functional, aldehyde groups (CHO, 1725 cm^-1^), ketones (C = O, 1715 cm^-1^) and carboxylic acids (COOH, 1,710 cm^-1^), [76]. In this study, a group of signals of difficult interpretation was observed on the 1,700–1,750 cm^-1^ regions of FTIR results (Fig 3) that could be associated with the aforementioned functional groups. Signal on 875 cm^-1^ correspond to ether or peroxide bonds. Presence of such band has been described after LDPE_oxo_ incubation with *P*. *ostreatus* for 45 days [77,78].

A decrease in TM1’s I*co* value was associated with loss of the C = O and C-O bonds [50], depicting the fungus assimilation for these functional groups (Table 1). Peroxidases generated free radicals on the PEBD chain, which can attack the plastic’s carbonated chains resulting in low molecular weight oxidized products, such a COOH, C-OH and C = O [50]. In this sense, *P*. *ostreatus* could have consumed these low molecular weight products, evidenced by decreased I*co* value. I*co* value decrease, suggesting microbial attack of oxidized chains, where C = O groups become transformed into C = OOH, after β-oxidation metabolism [79].

### Biochar production

The bio-product obtained from all fermentations carried out in microcosms at day 75 was converted into RM for BC production at 300°C/1h. Thermal transformation accounted for water, oxygen, nitrogen and carbon loss by volatilization, yet stable carbon or fixed fraction was concentrated. At 300°C moisture is lost and lignocellulose suffers thermal depolymerization processes, producing a carbon matrix with different degrees of aromaticity [80]. These results were associated with H/O and O/C ratios, which were low and suggested during thermal treatment water and oxygen were eliminated, and stable aromatic carbon was favored. Additionally, low O/C ratios also suggest BC surface was less polar than RM and tended to be less hydrophilic [81]. BC texture was porous, had greater surface contact and more homogenous particle size than RM. It has been reported that biomass thermal treatment can induce micro- and macro-pore formation, which is advantageous for microorganism colonization [82]. BC’s bee-hive like structure was characterized by SEM observations, which was formed at these temperatures when pine residues are employed, as has been demonstrated by using PB to produce BC at different temperatures. In their work they evidenced a bee-hive like structure, when BC was produced at 300° C under reduced oxygen conditions [80].

Following BC production, it was enriched with PSB here known as BC/PSB/I and its respective secondary culture (BC/PSB/SC). Despite BC’s low hydrophilicity, it was successfully moistened with the liquid biofertilizer, resulting in an increase in moisture percentage, organic matter and organic carbon content, which were contributed by bacterial liquid culture (Table 2) leading to an 81% increase in volatile carbon. On the contrary, fixed carbon decreased as a result of supplementation with other compounds rich in nitrogen and carbon, not associated with aromatic and amorphous carbon formed at 300°C.

An important result was obtained from element analysis, where an increase in hydrogen and oxygen content was observed, due to BC hydration. Therefore, molar ratios were starkly increased and solid biofertilizer became more hydrophilic. Furthermore, BC’s physical characteristics (hydrophilic surface, bee-hive structure, and micro-and macro-pore) allowed for an elevated PSB number to be immobilized (9.5 Log_10_ CFU g^-1^). No adverse effect was observed of BC on PSB. Moreover, when secondary culture was carried out an increase in approximately one logarithmic unit was observed. Additionally, during formulation process none of the morphotypes were eliminated.

*A*. *cepa* plants seeded at low doses on BC/PSB/SC (T1, T2 and T3), had greater plant biomass compared with those seeded on other substrates. Plant’s weights and heights were significantly higher at 5% BC + PSB (*p* = 0.0010 and *p* = 0.0023), followed by 2% and 1%, respectively.

Biochar’s properties as a microbial carrier were demonstrated by BC/PSB high PSB populations, microorganism biofilm arrangement on the biomaterial, and bacteria recovery from the substrate used for plant seeding in treatments including the biofertilizer. It is known adding PSB to different organic materials has an effect on bacterial community composition, phosphorous fractions, and organic acids produced [83]. *Kosakonia* sp., *Pseudomonas* sp., and *Serratia* sp., have the capacity to produce low molecular organic acids, such as oxalic and citric acid [84] that solubilize nutrients like P, Ca and K form diverse materials, protonate BC’s surface and decrease solubilized cation retention capacity, allowing *A*. *cepa* to acquire them from the substrate’s liquid phase. Several authors affirmed nutrients such as Ca^2+^, K and Mg^2+^, contained within bacterial culture or obtained as an organic nitrogen source product from bacteria metabolism, remain in the biofertilizer’s aqueous phase, and along with C and BC stimulate microorganism metabolism of different functional groups from the soil, which are then mixed with BC300 [85,86]. In the present study BYH was the organic nitrogen source. These nutrients can also be taken up by the onion’s root system.

In contrast to obtained materials at higher temperatures, BC roughness and hydrophilicity did not favor enzyme retention, such as phosphatases or β-glucosidases [87]. Therefore, nutrient mobility was augmented resulting in greater *Allium cepa* biomass, because of soil’s microbiota stimulation and PSB present in BC (BC/PSB). BC/PSB at low doses and soil mixture results on plant growth are worth highlighting, as they did not reduce priming effect reported by other authors [87]. On the contrary, it accounted for differences in plant growth between treatments and controls, as it supplied nutrients contained in OM as well as bacterial culture including rock phosphate (32% CaO), nitrates (0.2 mg L^-1^), extractable phosphorus (209 mg L^-1^) and water soluble phosphorus (41 mg L^-1^), [30,81,82].

Obtained results are important to help diminish the impact industrial activities have on the environment. The Holy Father Pope Francis in his encyclical letter “*LAUDATO SI* on care of our common home” (Chapter 1, No. 22) recognizes there is a “greater sensibility towards the environment and to protect nature, in addition to a growing concern for what is happening to our planet”. He makes reference the “throwaway culture” is a main problem associated with present planet contamination, and proposes to adopt a circular model of production to preserve resources, limit use of non-renewable resources, moderate consumption, in order to maximize efficiency of preservation by reusing and recycling.

Regarding resource preservation and reutilization the present work, results are an example of a circular production model at laboratory scale, since they integrate organic matter solid residue bioconversion from plant, brewer and service residues (plastics and napkins). By employing a lignolytic fungus *P*. *ostreatus* bioproducts were obtained with value added in comparison with raw material (Partially oxidized lignocellulosic biomass, biotransformed plastic) and CO_2_ captured with carbon dioxide trap.

The most abundant bioproduct obtained from these experiments was thermally transformed to obtain biochar, with physical and chemical properties and possible beneficial properties for soil, aiding in contaminant stabilization and carbon sequestration. Thus, enriching soil by providing sources of carbon and nutrients, retaining water, and reinforcing the soil’s microbial and enzyme activities.

Adding up all the benefits biochar by itself provides, it must be highlighted the biomaterial was enriched with PSB, which was evaluated at greenhouse scale on lettuce growth. Development of this fertilizer was twofold, first to biotransform agroindustrial residues, and second it could be used as a biological fertilizer.

### Conclusions

*P*. *ostreatus* under mesophilic and aerobic conditions at microcosm scale is promising for LCB and plasma treated LDPE_oxo_ simultaneous treatment. The designed system favored *P*. *ostreatus* growth, as it integrated a composition rich in lignocellulosic matter, substrate for the fungus’ lignolytic enzymes. In particular, LiP presented the highest enzyme activity at day 75, assisting in plasma treated LDPE_oxo_ sheet biotransformation. Both residues biotransformation was possible due to co-metabolism between substrates, influenced by lignolytic enzyme and biomass production. One obtained bioproduct was converted into a substrate for PSB immobilization, demonstrating a potential use as a solid organic biofertilizer.

## Supporting information

S1 Supplementary Material 2^3^ factorial design with three centrals points. (DOCX)

S1 Table 2^3^ factorial design with three central points for microcosm system filling mixture selection. (DOCX)

S2 Table Biomass colonization (%), Total Organic Carbon and Organic Matter 2^3^ factorial Design response variables (ANOVA). (DOCX)

S3 Table Enzymatic activities 2^3^ factorial design response variables (ANOVA). (DOCX)

S4 Table 2^3^ factorial design results for microcosm filling mixture selection. (DOCX)

## Abbreviations

ABTS: 2,2′-Azino-bis (3-ethylbenzothiazoline-6-sulfonic acid).
AFM: Atomic force microscopy
BC: Biochar (produced 300° C / 1 hour)
BC/PSB/I: BC + PSB initial
BC/PSB/SC: BC/PSB secondary culture (before 30°C /24 hours).
BYH: Brewing yeast hydrolysate
CFU: Colony-forming unit.
CM1: PB + CPN + BYH + *P*. *ostreatus*
CM2: PB + CPN + BYH + LDPE_oxo_
CM3: PB + CPN + BYH
CPN: Chopped paper napkins
EDS: Energy dispersive X-ray spectroscopy
FTIR: Fourier transform infrared spectroscopy
Ico: Carbonyl index
Iv: Vinyl index
Lac: Laccase
LCB: Lignocellulosic biomass.
LDPE_oxo_: Low density oxodegradable polyethylene
LiP: Lignin peroxidase
MnP: Manganese peroxidase
OM: Organic matter
PB: Pine bark
PO: *Pleurotus ostreatus*
PSB: Phosphate solubilizing bacteria
RM: Raw matter.
SCA: Static contact angle
SD: Standard deviation
SEI: Secondary electron image
SEM: Scanning electron microscopy
TM1: PB + CPN + BYH +LDPE_oxo_ + *P*. *ostreatus*
TOC: Total organic carbon
