# Supplementary material for: Simultaneous bioconversion of lignocellulosic residues and oxodegradable polyethylene by Pleurotus ostreatus for biochar production, enriched with phosphate solubilizing bacteria for agricultural use
Source: PLoS One. 2019 May 16;14(5):e0217100. doi: 10.1371/journal.pone.0217100 (PMC6521990; doi:10.1371/journal.pone.0217100)
Supplement: S1 Supplementary Material — (DOCX) [file pone.0217100.s001.docx]

**S1 Supplementary Material.** 2^3^ factorial design with three centrals points

To 90 mm Petri dishes containing agar-water (10 g bacteriological agar in 1,000 mL distilled water) 50 mm diameter circular segments were removed with a sterile scalpel, where different LCB dry plant matter were placed to be evaluated as filling mixtures (S4 Table). Three plasma treated LDPE_oxo_ sheets and 1 g moist *P. ostreatus* biomass were weekly hydrated with 5.3 mL nutrient solution and redox mediator (0.625 gL^-1^glucose, 1.5 gL^-1^ CuSO_4_, 0.1 mM ABTS, 2 gL^-1^KH_2_PO_4_, 0.050 gL^-1^NH_4_Cl, 0.5 gL^-1^MgSO_4_·7H_2_O, 0.1 gL^-1^ CaCl_2_·2H_2_O and 10 mL trace solution: 0.5 gL^-1^MnSO_4_, 0.1 gL^-1^FeSO_4_·7H_2_O, 0.1 gL^-1^ ZnSO_4_·7H_2_O at pH 5.7). Petri dishes were incubated at 28 ºC for 10 days. Colonization percentage was determined (6), extraction for Lac, MnP and LiP enzyme evaluation and TOC and total OM percentage assessment.
