# Supplementary material for: Simultaneous bioconversion of lignocellulosic residues and oxodegradable polyethylene by Pleurotus ostreatus for biochar production, enriched with phosphate solubilizing bacteria for agricultural use
Source: PLoS One. 2019 May 16;14(5):e0217100. doi: 10.1371/journal.pone.0217100 (PMC6521990; doi:10.1371/journal.pone.0217100)
Supplement: S1 Table — (DOCX) [file pone.0217100.s002.docx]

**S1 Table**. 2^3^ factorial design with three central points for microcosm system filling mixture selection.

| Coded matrix | | | | Nominal matrix | | |
| --- | --- | --- | --- | --- | --- | --- |
| T | PB (g) | CPN (g) | BYH (g) | PB (g) | CPN (g) | BYH (g) |
| 1 | -1 | -1 | -1 | 1 | 0.5 | 1 |
| 2 | +1 | -1 | -1 | 5 | 0.5 | 1 |
| 3 | -1 | +1 | -1 | 1 | 5.5 | 1 |
| 4 | +1 | +1 | -1 | 5 | 5.5 | 1 |
| 5 | -1 | -1 | +1 | 1 | 0.5 | 2 |
| 6 | +1 | -1 | +1 | 5 | 0.5 | 2 |
| 7 | -1 | +1 | +1 | 1 | 5.5 | 2 |
| 8 | +1 | +1 | +1 | 5 | 5.5 | 2 |
| 9 | PC | PC | PC | 3 | 3 | 1.5 |
| 10 | PC | PC | PC | 3 | 3 | 1.5 |
| 11 | PC | PC | PC | 3 | 3 | 1.5 |
