# Supplementary material for: Simultaneous bioconversion of lignocellulosic residues and oxodegradable polyethylene by Pleurotus ostreatus for biochar production, enriched with phosphate solubilizing bacteria for agricultural use
Source: PLoS One. 2019 May 16;14(5):e0217100. doi: 10.1371/journal.pone.0217100 (PMC6521990; doi:10.1371/journal.pone.0217100)
Supplement: S2 Table — (DOCX) [file pone.0217100.s003.docx]

**S2 Table**. Biomass colonization (%), Total Organic Carbon and Organic Matter 2^3^ factorial Design response variables (ANOVA)

| **Biomass Colonization (%)** | | | | **Total Organic Carbon total (%)** | | | | **Organic Matter (%)** | | | |
| --- | --- | --- | --- | --- | --- | --- | --- | --- | --- | --- | --- |
| **Factor** | ***p value*** | **Contribution**  **(%)** | **Stand. Effect** | **Factor** | ***p value*** | **Contribution**  **(%)** | **Stand. Effect** | **Factor** | ***p value*** | **Contribution**  **(%)** | **Stand. Effect** |
| Model | **0.0096** |  | +86 | Model | **0.0122** |  | +36.5 | Model | **0.013** |  | 63.5 |
| A: CP | 0.057 | 1.8 | +1.67 | A: CP | **0.0116** | 11.5 | +3.75 | A: CP | **0,013** | 11.078 | 6.25 |
| B: SP | **0.043** | 2.4 | -1.88 | B: SP | **0,0213** | 6.21 | +2.75 | B: SP | **0.023** | 6.4 | 4.75 |
| C: HLC | **0.0031** | 38 | +7.37 | C: HLC | 0.345 | 0.20 | -0.50 | C: HLC | 0.41 | 0.159 | -0.75 |
| AB | **0.0124** | 9,2 | -3.6 | AB | 0.134 | 0.821 | +1 | AB | 0.11 | 1.13 | 2.0 |
| AC | 0.45 | 0.098 | -0.37 | AC | **0.0024** | 55.9 | +8.25 | AC | **0.028** | 55.6 | 14 |
| BC | **0.023** | 4.82 | +2.63 | BC | 0.0503 | 2.51 | -1.75 | BC | 0.055 | 2.55 | -3.0 |
| ABC | **0.0041** | 28 | +6.37 | ABC | **0.0134** | 0.82 | -1 | ABC | 0.14 | 0.868 | -1.75 |
| Curvature F-value | 0.078 | 14 |  | Curvature F-value | 0.0063 | 21 |  | Curvature F-value |  | 22 |  |

In **bold** model’s significance and significant factors within the model (*p* < 0.05).
