# Supplementary material for: Simultaneous bioconversion of lignocellulosic residues and oxodegradable polyethylene by Pleurotus ostreatus for biochar production, enriched with phosphate solubilizing bacteria for agricultural use
Source: PLoS One. 2019 May 16;14(5):e0217100. doi: 10.1371/journal.pone.0217100 (PMC6521990; doi:10.1371/journal.pone.0217100)
Supplement: S3 Table — (DOCX) [file pone.0217100.s004.docx]

**S3 Table.** Enzymatic activities 2^3^ factorial design response variables (ANOVA)

| **Laccase Activity (U Kg^-1^)** | | | | **MnP Activity (U kg^-1^)** | | | | **LiP Activity (U kg^-1^)** | | | |
| --- | --- | --- | --- | --- | --- | --- | --- | --- | --- | --- | --- |
| **Factor** | ***p value*** | **Contribution**  **(%)** | **Stand. Effect** | **Factor** | ***p value*** | **Contribution**  **(%)** | **Stand. Effect** | **Factor** | ***p value*** | **Contribution**  **(%)** | **Stand. Effect** |
| Model | **0.0070** |  | +200 | Model | **0.0273** |  | +80.5 | Model | 0.068 |  | +6802 |
| A: CP | **0.0060** | 13.3 | +49.88 | A: CP | 0.0932 | 3.16 | +11.5 | A: CP | 0.419 | 0.74 | -369 |
| B: SP | **0.0431** | 1.76 | +18.13 | B: SP | **0.0049** | 69.2 | +53.7 | B: SP | 0.129 | 4.55 | +914 |
| C: HLC | **0.0056** | 14.2 | -51.63 | C: HLC | 0.816 | 0.023 | -1 | C: HLC | 0.403 | 0.808 | +385 |
| AB | **0.0059** | 13.5 | +50.3 | AB | 0.077 | 3.89 | +12.7 | AB | 0.133 | 4.4 | +898 |
| AC | **0.0168** | 4.7 | +29.6 | AC | 0.108 | 2.64 | +10.5 | AC | 0.051 | 13.2 | +1556 |
| BC | **0.0024** | 33.3 | +78.8 | BC | 0.077 | 3.89 | -12.7 | BC | **0.020** | 34.5 | +2519 |
| ABC | 0.4356 | 0.114 | +4.63 | ABC | **0.0489** | 3.30 | +11.75 | ABC | **0.0452** | 12.9 | +1540 |
| Curvature F-value | 0.0043 | 18.7 |  | Curvature F-value | 0.025 | 13.1 |  | Curvature F-value | 0.025 | 27 |  |

In **bold** model’s significance and significant factors within the model (*p* < 0.05).
