# Supplementary material for: Simultaneous bioconversion of lignocellulosic residues and oxodegradable polyethylene by Pleurotus ostreatus for biochar production, enriched with phosphate solubilizing bacteria for agricultural use
Source: PLoS One. 2019 May 16;14(5):e0217100. doi: 10.1371/journal.pone.0217100 (PMC6521990; doi:10.1371/journal.pone.0217100)
Supplement: S4 Table — (DOCX) [file pone.0217100.s005.docx]

**S4 Table**. 2^3^ factorial design results for microcosm filling mixture selection.

| T | Colonization (%) | Lac U kg^-1^ | MnP U kg^-1^ | LiP U kg^-1^ |
| --- | --- | --- | --- | --- |
| 1 | 72 **^e^** | 338 **^b^** | 15 **^e^** | 9306 **^b^** |
| **2** | **96 ^b^** | **287 ^c^** | **15 ^e^** | **6738 ^c^** |
| 3 | 83 **^d^** | 125 **^d^** | 146 **^b^** | 7380 **^b^** |
| 4 | 67 **^e^** | 257 **^c^** | 150 **^b^** | 2246 **^d^** |
| **5** | **95 ^b^** | **27 ^e^** | **41 ^d^** | **5006 ^c^** |
| 6 | 92 **^c^** | 76 **^e^** | 36 **^d^** | 2503 **^d^** |
| 7 | 91 **^c^** | 111 **^d^** | 74 **^c^** | 6995 **^c^** |
| **8** | **99^a^** | **380 ^a^** | **167 ^a^** | **14248 ^a^** |

In bold best treatments with significant differences are highlighted *p* < 0.05. Letters represent Tukey homogeneous subsets. a* corresponds to the best treatment, followed in order by b, c, d and e.
